# High quality genome assemblies of African cattle breeds using PacBio HiFi sequencing

**DOI:** 10.1038/s41597-026-07589-2

**Published:** 2026-06-06

**Authors:** Isidore Houaga, Meenu Bhati, Zabron Nziku, Athumani Nguluma, Gilbert Msuta, Mapholi Ntanganedzeni, Lucky T. Nesengani, Moses Ogugo, Duhamel C. Y. Sagbo, Loukaïya Zorobouragui, Mariano B. Y. Boco, Appolinaire Djikeng, James G. D. Prendergast, Gregor Gorjanc, Hannes Becher

**Affiliations:** 1 The Roslin Institute, Royal (Dick) School of Veterinary Studies, University of Edinburgh, Easter Bush Campus, Midlothian, EH25 9RG UK; 2 Centre for Tropical Livestock Genetics and Health, Easter Bush, Midlothian, EH25 9RG UK; 3 Department of Agriculture and Animal Health, College of Agriculture and Environmental Sciences, The University of South Africa, Cnr Justice Mahomed & Steve Biko Streets, PO Box 392 Pretoria, South Africa; 4 Tanzania Livestock Research Institute (TALIRI), Dodoma, Tanzania; 5 Department of Animal, Aquaculture, and Range Sciences, Sokoine University of Agriculture, Morogoro, Tanzania; 6 The International Livestock Research Institute, PO Box 30709 Nairobi, Kenya; 7 African Institute of Data Driven Animal and Plant Breeding and Genetics (DATAGENES-AFRICA), Aplahoue, Benin; 8 Laboratory of Ecology, Health and Animal Production (LESPA), Faculty of Agronomy, University of Parakou, Parakou, Benin; 9 Ferme d’Élevage de Samiondji BP: 50 Covè, Covè, Benin; 10 Present Address: The International Livestock Research Institute, PO Box 30709 Nairobi, Kenya

**Keywords:** Genome, Genome informatics

## Abstract

Africa has a uniquely rich cattle diversity of ~150 breeds comprising the *Bos taurus indicus* sub-species, *Bos taurus taurus*, and their crosses. These represent ~23% of the global cattle population. However, high quality, representative assemblies are limited for African cattle and especially for indicine breeds. Here we built high quality *de novo* assemblies for five important African indigenous cattle breeds using PacBio HiFi sequencing: Lagune (*Bos taurus taurus)*, Gudali, Iringa Red and Singida White (*Bos taurus indicus)*, and Mpwapwa *(Bos taurus taurus x Bos taurus indicus*). These new assemblies are the most contiguous and complete African cattle assemblies produced so far, with genome sizes of 3.25–3.36 Gb, contiguity N50s ranging from 83.59 Mb to 97.87 Mb and scaffold N50s from 100.30 Mb to 113.37 Mb. BUSCO genome completeness scores were also higher than 99.68%, indicative of highly contiguous assemblies. These improved and highly contiguous genome assemblies are consequently a valuable resource for future African and global livestock genomic studies.

## Background & Summary

Cattle play important roles globally and in Africa. Providing meat and milk, they are a significant source of nutrition and livelihood to many African societies. They are also used in agriculture as a source of traction and manure. Further, cattle are of great societal importance, including during birth, marriage, death, or traditional ceremonies, and are a representation of prestige and power^1,2^. Since their domestication around 10,000 years ago, hundreds of distinct cattle breeds have been developed, characterised by high variation in physical appearance, productivity levels, environmental adaptation and disease tolerance^3^. Africa has a uniquely rich cattle diversity with about 150 known cattle breeds or populations comprised of *Bos taurus taurus*, *Bos taurus indicus*, and their crosses^4^. These account for up to 23% of the global cattle population^5^, compared to only 8% for European cattle.

Despite the high diversity of African cattle, there is a conspicuous lack of high quality and representative assemblies, particularly for *Bos taurus indicus* breeds. Multiple introductions and migrations of both taurine and indicine cattle into Africa, combined with diverse environments, pathogens, and cultures have resulted in high genetic diversity among its cattle breeds^6,7^. However, this diversity is inadequately represented by currently published reference genomes, which comprise the Asian-derived breed Brahman^8^ and the European breeds Angus^8^ and Hereford^9^. A recent study generated the first African cattle genome assemblies for the West African Taurine N’Dama and an East African stabilized cross between indicine and taurine breeds, called Ankole. This study identified an extra 116.1 Mb (4.2%) of sequence absent from the current Hereford reference sequence^9^ and consequently inaccessible in previous studies^6^. However, the new assemblies for N’Dama and Ankole had modest contiguities, N50 of 10.7–18.6 Mb. Gene content completeness was 92.6%–93.1%, using Benchmarking Universal Single-Copy Orthologs (BUSCO), suggesting that more high quality and representative assemblies are needed for African cattle breeds. Such high quality and representative assemblies are expected to improve read mapping, allelic biases, structural variant calling, as well as downstream genomic analyses such as detecting signatures of selection^6,10^.

**Fig. 1:**
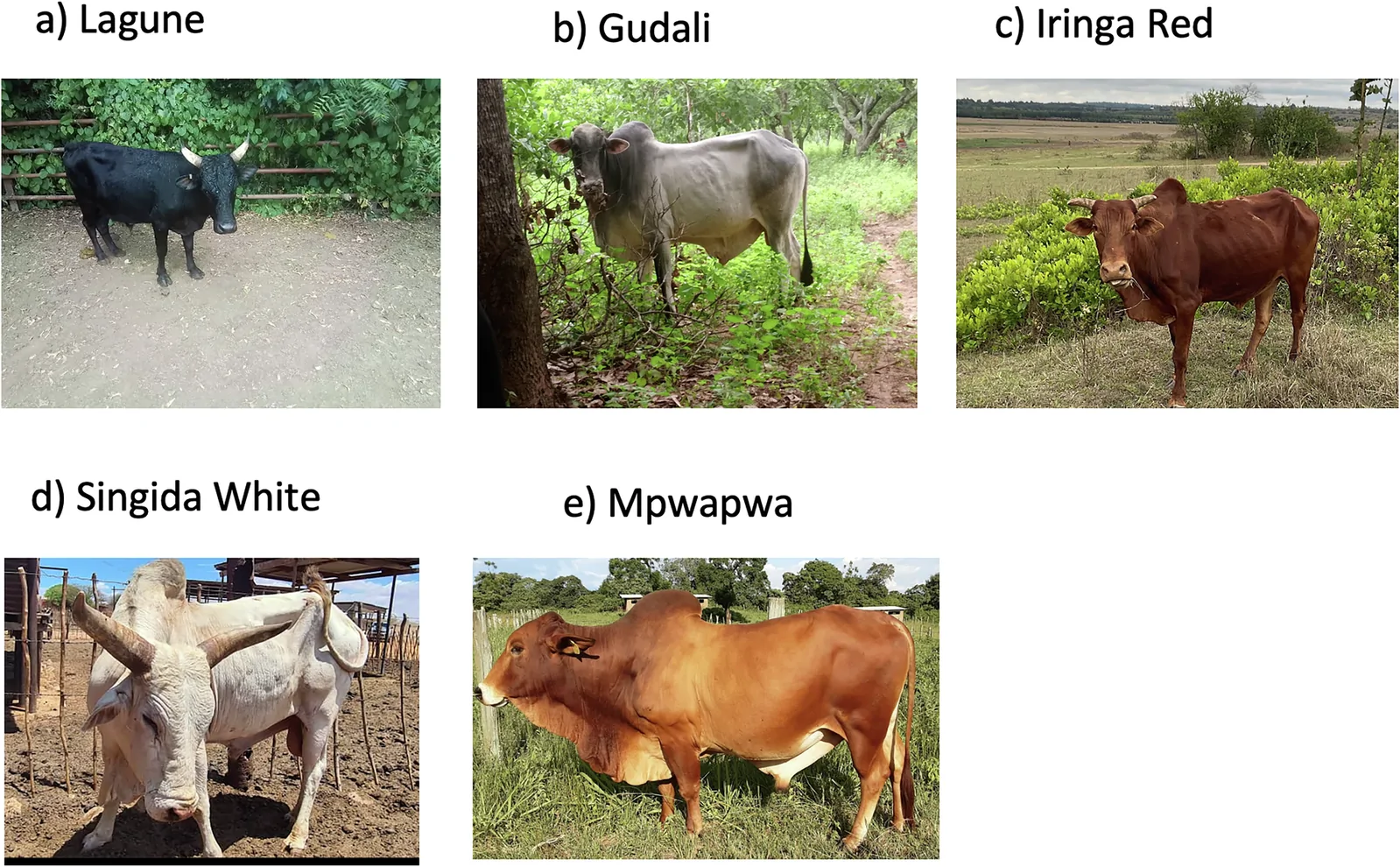
Pictures of the five sequenced cattle breeds: (**a**) Lagune (Bull 511), (**b**) Gudali, (**c**) Iringa Red, (**d**) Singida White and (**e**) Mpwapwa (Bull NAIC 906).

In this study, we address the current lack of high-quality reference genomes for African cattle breeds. Using PacBio HiFi sequencing data, we generated state-of-the-art *de novo* genome assemblies for five important African indigenous cattle breeds. These are Lagune (*Bos taurus taurus*), Gudali, Iringa Red and Singida White (all three *Bos taurus indicus*), and Mpwapwa (*Bos taurus taurus x Bos taurus indicus*). The five breeds are shown in Fig. 1. The Lagune and Gudali cattle breeds were sampled in the Republic of Benin, West Africa, while Iringa Red, Singida White and Mpwapwa cattle breeds were sampled in Tanzania, East Africa. **Lagune** is a small hardy breed known for its adaptability to humid tropical climates and resistance to diseases such as trypanosomiasis making it valuable for meat production in tsetse fly-infested regions^11^. **Gudali** is a robust breed originating from West and Central Africa, known as an excellent dairy producer. It is also known for its strength and adaptability to semi-arid regions and is used for draft purposes^12^. **Iringa Red** is native to Tanzania, prized for its drought resistance and ability to thrive in challenging environments, making it useful for meat production and agricultural work^13^. **Singida White** is a Tanzanian indigenous breed well adapted to harsh environments, known for its use in meat production and as farmer-preferred draft animals in rural areas^14^. **Mpwapwa** is a dual-purpose (milk and beef) breed developed in Tanzania, bred for resilience in tropical climates and resistance to common diseases, enhancing food security in rural communities^13^. The Mpwapwa, Iringa Red and Singida White breeds have never been subject to any whole-genome sequencing study.

Our new haplotype-resolved assemblies are the most contiguous and complete African cattle assemblies produced so far with genome size ranging from 3.25 to 3.36 Gb and with heterozygosity ranging between 0.37% (Lagune) and 0.68% (Iringa Red). Contiguity N50 ranged from 83.59 Mb (Iringa Red) to 97.87 Mb (Lagune) and scaffold N50 from 100.30 Mb (Iringa Red) to 113.37 Mb (Lagune). BUSCO genome completeness as estimated by *Compleasm* using the mammalian set (n = 9,226) was higher than 99.68% for all assemblies. Assembly accuracies, assessed with Merqury, exceeded QV 67 (99.9999%). Across the five indigenous cattle genomes, an average of 1.84 Gb of repeat sequences were identified, representing approximately 56% of each genome, with retroelements being the most abundant repeat class. These improved and highly accurate genome assemblies will provide valuable resources for African and global genomic studies of cattle and livestock in general.

## Methods

### Ethics statement

The study was approved by the Tanzania Livestock Research Institute Ethical Clearance Committee (Reference number: TLRI/CC.21/024). The blood collection was carried out by trained local veterinarians, according to the approved institutional guidelines. We obtained consent from all farmers before collecting blood samples.

### Animal sampling

In this study, a Lagune bull (ID:511, born on 20/01/2020) was selected at the State-owned Samiondji Breeding Farm (07°26′30″ N, 02°22′59″ E) in Zagnanado Commune, in the Zou Department, southern-central Benin. A Gudali cow was selected from a smallholder farmer at Kasuala (8°53′18.96″N 2°44′56.796″E), Commune of Tchaourou, in the Borgou Department, northern-east Benin. The Iringa Red bull was selected from a smallholder farmer at Ibumila (8°3′28.17″ S, 35° 33′47.65″ E), Iringa Region, Central Tanzania. The Singida White cattle breed cow (ID:01) was selected at the Tanzania Livestock Research Institute, research station located in Kongwa (6°12′0″ S, 36°25′0″ E), Dodoma region, Tanzania. A Mpwapwa cattle breed bull (NAIC 906, born on 26/06/2013) was selected at the Tanzania National Artificial Insemination Centre (3°22′12″ S, 36°50′09″ E) located in the Usa River, Arusha, Northen Tanzania. Blood samples were collected from the jugular vein into a labelled 5 ml EDTA vacutainer tubes and immediately transferred to the laboratory in a cool box containing icepack and stored at −20 °C until DNA extraction.

### DNA extraction and PacBio long-reads Sequencing

The blood samples were allowed to thaw for 30 minutes at room temperature. High Molecular Weight (HMW) DNA was extracted from 200 μL bovine whole blood using the Pacific Biosciences (PacBio) HMW DNA extraction from mammalian cells – Nanobind® tissue kit (102-302-100) following manufacturer’s instructions. The PacBio SMRTBell® prep kit 3.0 was used for library preparation and the Revio polymerase kit 1.0 (Pacific Biosciences) was used for loading the libraries onto the sequencer. The samples were sequenced at 225 pM on the PacBio Revio platform at Edinburgh Genomics using two Revio SMRT Cells per sample.

### Genome assembly

De novo assembly was run with the 53–72X coverage obtained from each sample using hifiasm (v0.19.9)^15^ with default parameter except for trimming the first 20 base pairs. The shell scripts containing the exact series of commands used to generate the assemblies is presented in Supplementary 1. The assemblies’ contigs were scaffolded using the recently published Hereford reference cattle genome (ARS-UCD2.0) using RagTag (v 2.1.0)^16^ without the correction step because of the high quality of our assemblies. Despite the large contig N50 values, the use of a synteny-based scaffolding approach based on a reference assembly from a different cattle breed represents a potential limitation, as reference assembly errors could be propagated and breed-specific structural rearrangements may remain undetected. Contaminant contigs identified and flagged during the National Center for Biotechnology Information (NCBI) submission process were removed from the final genome assemblies. These contigs were all unplaced and did not correspond to autosomal, sex chromosomal, or mitochondrial sequences, ensuring that only high-confidence genomic regions were retained.

### Repeat sequences and annotation

Repeat annotation of the five African cattle genome assemblies and ARS UCD2.0 was performed using RepeatMasker (v4.1.5)^17^ with established repeat libraries, including Dfam (v3.8)^18^ and the RepBase RepeatMasker Edition (20181026) and tandem repeats with TRF (v4.09.1). De novo repeat identification from RepeatModeler (v2.0.5)^19^ was not integrated due to the high coverage provided by these curated libraries, resulting in only 0.01% of unidentified repeats.

## Data Records

The raw sequences were submitted to the NCBI and assigned accession number SRA (SRP586484) data of PRJNA1197437 with accession numbers of SRX28862361, SRX28862362, SRX28862363, SRX28862364 and SRX28862365 respectively for Lagune, Gudali, Mpwapwa, Iringa Red and Singida White^20^. Each sample is associated with the following experiment (SRX), read set (SRR), and library:

Lagune: SRX28862361 (Library: LAG), SRR33634532

Gudali: SRX28862362 (Library: GUD), SRR33634531

Mpwapwa: SRX28862363 (Library: MPW), SRR33634530

Iringa Red: SRX28862364 (Library: IRR), SRR33634529

Singida White: SRX28862365 (Library: SIW), SRR33634528

The five scaffolded primary genome assemblies were submitted to NCBI under the BioProject PRJNA1197437 and assigned the accession number JBNBOL000000000^21^. The Lagune, Gudali, Mpwapwa, Iringa Red and Singida White genome assemblies were respectively assigned BioSample SAMN45804670, SAMN45804671, SAMN45804672, SAMN45804673 and SAMN45804674)^21^.

### Data overview

The sequencing yielded a total of 8.2–12.6 M reads and 160–219 Gbp, corresponding to a genomic coverage depth of approximately 53–72X. The summary statistics of the sequencing reads are presented in Table 1.

**Table 1:** Summary statistics of the raw sequencing data.

Cattle breed	Subpecies	Country/Region	Sex	HiFi Yield (Gb)	Mean HiFi RL (bp)	Median QV	Coverage
**Lagune**	*Bos taurus*	Benin/West-Africa	♂	218	19,476	32	72×
**Gudali**	*Bos indicus*	Benin/West-Africa	♀	219	19,905	34	73×
**Iringa Red**	*Bos indicus*	Tanzania/East-Africa	♂	160	19,479	32	53×
**Singida White**	*Bos indicus*	Tanzania/East-Africa	♀	190	15,147	33	63×
**Mpwapwa**	*Bos taurus × Bos indicus*	Tanzania/East-Africa	♂	204	18,195	33	68×

RL: Read Length.

## Technical Validation

### African cattle genome assemblies and quality assessment

**Fig. 2:**
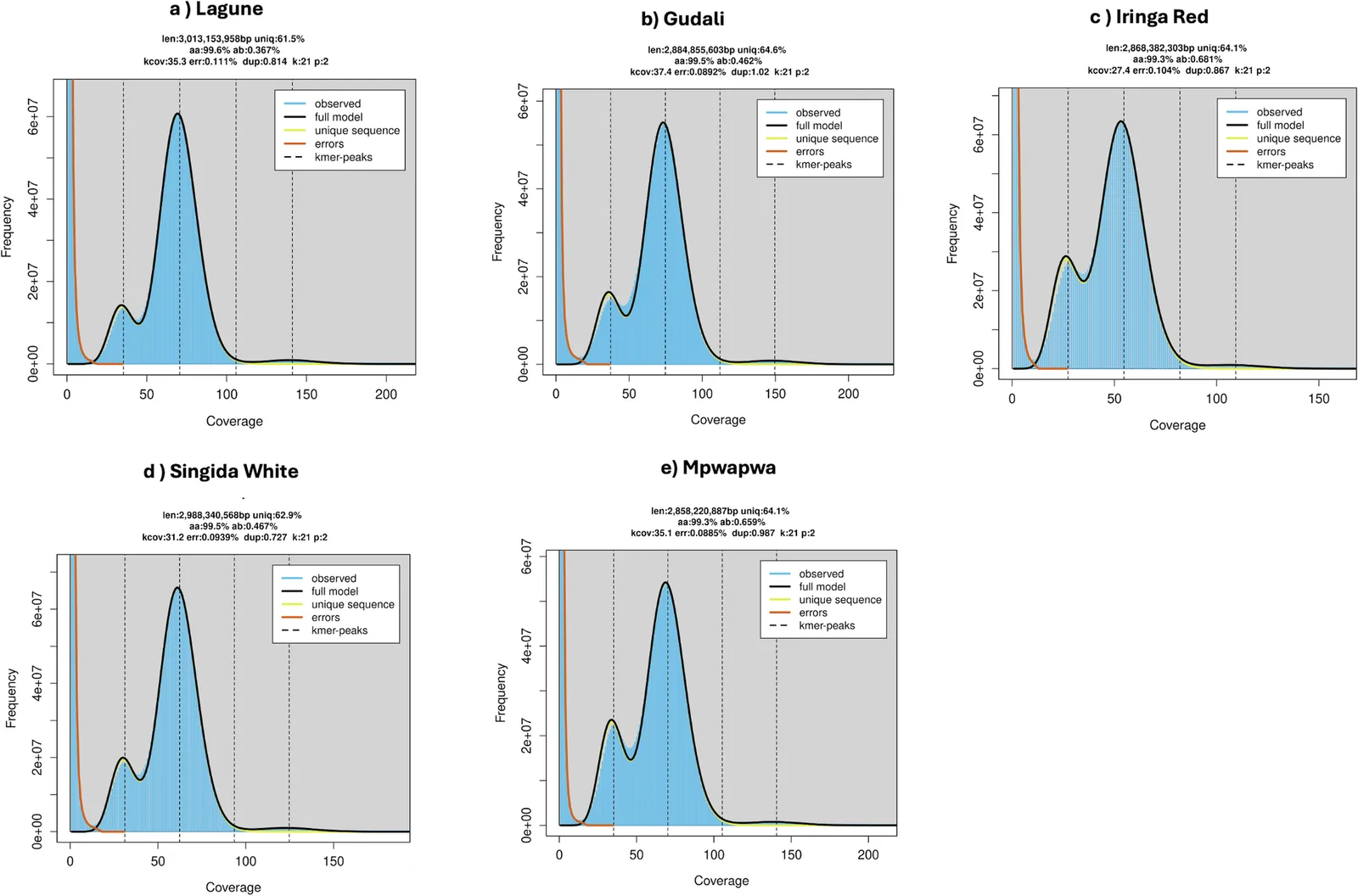
GenomeScope profiles for five novel African cattle genome assemblies. The estimated genome size ranged between 2.88 (Gudali) and 3.01 Gb (Lagune) with heterozygosity between 0.36 (Lagune) and 0.68% (Iringa Red).

**Fig. 3:**
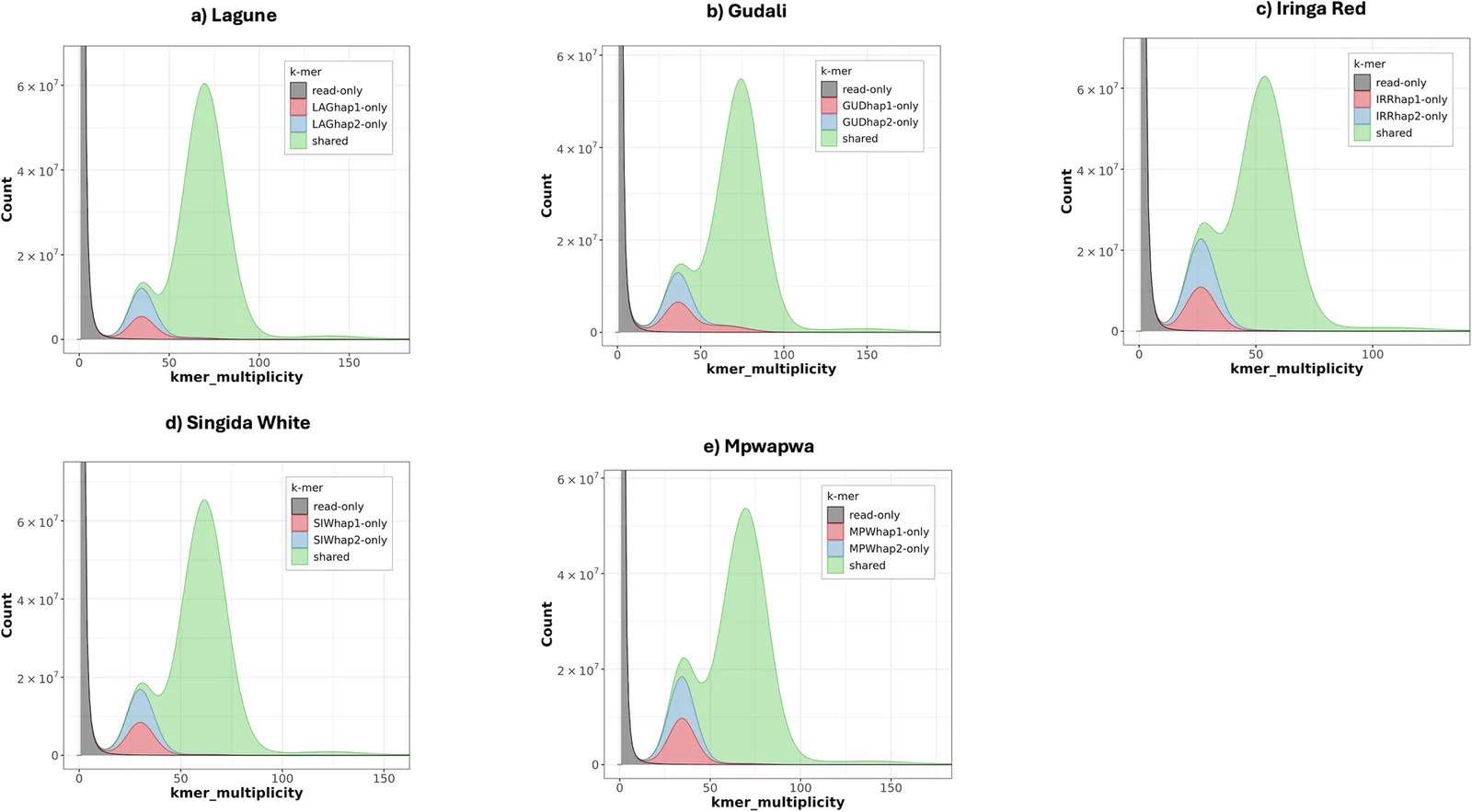
Merqury k-mer spectrum copy number (CN) plots for five haplotype-resolved African cattle genome assemblies. K-mers shared between raw sequencing reads and haplogenomes are shown in green, those private to one haplogenome are shown in blue and red. K-mers absent from the assembly are shown in grey.

We used FastQC (v0.12.1, https://www.bioinformatics.babraham.ac.uk/projects/fastqc/) to assess the quality of the sequencing reads using default parameters, and summarised the results using multiQC^22^. This did not result in any evidence for sequencing adapter contamination. After assembling the genomes, we employed the GenomeScope (v 2.0)^23^ for genome profiling using 21-mer counts generated by Meryl (v1.2; https://github.com/marbl/meryl) from the HiFi sequencing reads, see Fig. 2. We evaluated the quality of the primary genome assembly using Merqury (v1.1)^24^, see Fig. 3. The spectra are used to compare k-mers from the sequencing reads with those found in the haplotype-resolved genome assemblies, providing insights into assembly completeness, missing data, and errors. Across all breeds, the k-mer spetra show two prominent peaks as expected for diploids individuals with some degree of heterozygosity. In each plot, shared k-mers in green dominate in the right-hand diploid 2x peak, whereas haplotype-specific k-mers shown in red and blue dominate the left-hand haploid 1x peak. K-mers present in the sequencing data but absent from the final assemblies are shown in grey. These are largely of low multiplicity, at the very left of each plot, indicating that sequencing errors and contamination were not erroneously incorporated and that the assemblies are highly complete. The completeness and integrity of the genome assemblies were further evaluated using the BUSCO implemented in compleasm^25^ using the mammalian dataset “mammalia_odb10” (n = 9226). The evaluation found that 99.68% (9195) to 99.93% (9220) of the core mammalian genes were present in the African cattle genomes, including 98.46 to 98.65% single-copy, 1.19% to 1.47% duplicated, 0.03% fragmented and 0.03% to 0.29% missing genes from the mammalian data set (Table 2). The k-mer databases were used to calculate the overall quality values of the assemblies using Merqury^18^. The assemblies had high accuracy with QV 67.5-QV 71.7 (>99.9999%, Table 2). These confirmed the completeness of the five African cattle breeds reported in this manuscript. The QUAST^26^ software (v5.2.0) calculated that the five new assemblies cover 95.2% (GUD and SIW), 95.6% (IRR), 95.8% (MPW) and 96.4% (LAG) of the ARS-UCD2.0 (the most recent Hereford genome) higher than the reported 93.9% and 94.0% of the ARS-UCD1.2 Hereford genome covered respectively by the African N’Dama and Ankole cattle breeds^6^. The genome lengths ranged from 3.25 Gb (GUD) to 3.36 Gb (SIW) with 340 (MPW) to 622 (SIW) contigs. The contig N50 ranged from 83.59 Mb (Iringa Red) to 97.87 Mb (Lagune) higher than 10.70 Mb and 18.60 Mb previously reported for the N’Dama and the Ankole cattle breeds respectively^6^. The scaffolded assemblies had 222 (GUD) to 446 (SIW) scaffolds in total with scaffold N50 of 100.30 Mb (Iringa Red) to 113.37 Mb (Lagune) as shown in Table 2. The scaffold N50 of Lagune (113.37), Singida White (108.17) and Mpwapwa (105.95 Mb) are higher than the values of 104.80 Mb and 84.50 Mb reported for the N’Dama and Ankole genomes, respectively^6^. These statistics and the above quality assessment metrics confirm that our new assemblies are the most contiguous and complete African cattle assemblies produced so far.

**Table 2:** Genome assembly statistics of five African cattle breeds.

Statistics	Lagune	Gudali	Iringa Red	Singida White	Mpwapwa
**Raw primary assemblies as from** ***hifiasm***^15^					
Consensus accuracy	QV67.5	QV69.6	QV68.7	QV68.9	QV71.7
Genome size (bp)	3,338,004,939	3,252,386,270	3,302,534,872	3,356,428,914	3,296,393,950
GC content (%)	44.01	43.95	44.09	44.26	44.09
Heterozygosity	0.37	0.46	0.68	0.47	0.66
**#Contigs**	456	392	414	622	340
Average contig length (bp)	7,320,186	8,296,903	7,977,137	5,396,187	9695276
Contig N50	97,870,685	94,506,920	83,585,658	91,475,713	96,250,906
Contig L50	14	14	15	15	14
Largest contig	173,713,701	173,964,285	161,906,315	172,444,127	164,144,583
Smallest contig	15,073	16,340	5,341	11,215	16,271
**Clean scaffolded primary genome assemblies deposited at GenBank**					
**#Scaffolds**	292	222	308	446	302
Total scaffold length (bp)	3,327,642,104	3,239,885,270	3,296,547,564	3,345,996,902	3,295,703,406
Average scaffold length (bp)	11,396,034	14,594,077	10,703,076	7,502,235	10,912,925
scaffold N50	113,365,931	100,524,608	100,300,525	108,170,087	105,951,773
scaffold L50	13	13	13	13	13
Largest scaffold	173,713,701	173,964,285	161,906,315	172,444,127	166,841,832
Smallest scaffold	15,073	16,340	20,870	11,215	16,271
ARS-UCD2.0 Genome fraction (%)	96.4	95.2	95.6	95.2	95.8
**# Completeness** (Compleasm), library: mammalia_odb10 (n = 9,226)	9,220 (99.93%)	9,220 (99.94%)	9,195 (99.68%)	9,220 (99.93%)	9,220 (99.93%)
Complete and Single copy	9,100 (98.63%)	9,101 (98.65%)	9,085 (98.47%)	9,084 (98.46%)	9,097 (98.60%)
Complete and duplicated	120 (1.3%)	119 (1.29%)	110(1.19%)	136 (1.47%)	123 (1.33%)
Fragmented	3 (0.03%)	3 (0.03%)	4 (0.04%)	3 (0.03%)	3 (0.03%)
Missing	3 (0.03%)	3 (0.03%)	27 (0.29%)	3 (0.03%)	3 (0.03%)

### Genomic relationships

We generated a dendrogram using Mashtree^27^ by estimating Mash distances between our assemblies and publicly available genomes from NCBI. The publicly available assemblies include outgroups (Human-GCA000001405.29 GRCh38.p14, Goat-GCA 001704415.2 ARS1.2, Sheep-ARS-UI Ramb v3.0, African Buffalo-GCA 902825105.1 and Water buffalo-GCF 019923935. 1 NDDB SH 1); *Bos indicus* (GCA_026262515.1 ASM2626251v1, GCA_026262465.1 ASM2626246v1, GCA 030269505. 1 ASM3026950v1, GCA 003369695.2 Brahman 1 and GCA 000247795.2 1.0); *Bos indicus × Bos taurus* (Ankole GCA_905123885) and *Bos taurus* (NDama GCA_905123515, Jersey GCA_021234555.1 ARS, GCA 021347905.1 ARS Holstein-Friesian 1, GCA 003369685.2 UOA Angus 1, Hereford ARS-UCD2.0, GCA 028973685.2 SNU Hanwoo 2.0*, GCA 040286185.1 ASM4028618v1* and GCA 018282465.1 ARS Simm1.0). We generated a dendrogram using Mashtree by estimating Mash distances between our assemblies and publicly available genomes from National Center for Biotechnology Information.

**Fig. 4:**
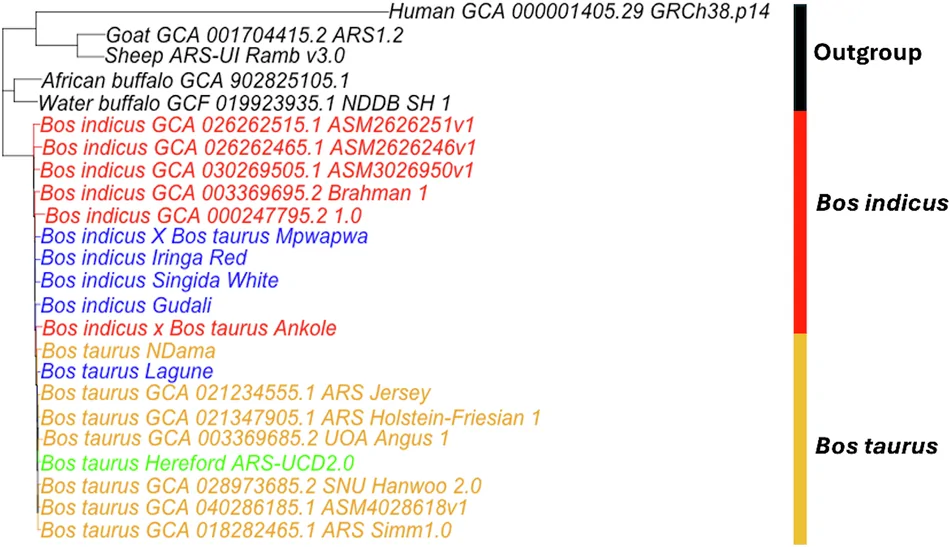
Genome similarities between new African cattle assemblies and publicly available assemblies. The new African assemblies generated here are in blue.

The genome-wide similarities between the new African cattle assemblies (blue) and publicly available assemblies are shown in Fig. 4. The new African taurine assembly (Lagune) was closely related to the African N’Dama taurine. The new assemblies of indicine origin and Bos indicus x Bos taurus crossbred also clustered together.

### Repeat sequences and annotation

The repeat content across the five African cattle genome assemblies is presented in Table 3. The total proportion of masked bases ranged from 55.7% to 56.9% (Singida White: 56.9%; Gudali: 55.7%), which is substantially higher by approximately eight percentage points than the ~49% reported for the ARS-UCD2.0 Bos taurus reference genome. This increase is driven primarily by satellite DNA, a major component of centromeric and pericentromeric regions, and likely reflects differences in assembly and post-processing strategies particularly the more complete retention of tandemly repeated centromeric regions in the larger non-reference genomes (~3.3 Gb vs 2.77 Gb).

**Table 3:** Repeat sequences contents across five African cattle breeds.

Main	subtype	Lagune	Gudali	Iringa Red	Singida White	Mpwapwa	ARS UCD2.0
Retroelements		1,286,930,678 (38.7%)	1,260,581,845 (38.9%)	1,266,327,947 (38.4%)	1,235,053,136 (37.9%)	1,275,256,348 (38.7%)	1218049094 (44.0%)
	LINEs	777,966,277 (23.4%)	761,124,491 (23.5%)	765,251,253 (23.2%)	746,988,345 (22.9%)	775,394,625 (23.5%)	765105434 (27.6%)
	LTR	184,078,941 (5.5%)	178,101,720 (5.5%)	179,680,343 (5.5%)	174,600,597 (5.4%)	176,345,576 (5.4%)	135029091 (4.9%)
	PLEs	57,571 (0.0%)	55,168 (0.0%)	54,606 (0.0%)	53,590 (0.0%)	54,058 (0.0%)	55936 (0.0%)
	SINEs	324,885,460 (9.8%)	321,355,634 (9.9%)	321,396,351 (9.7%)	313,464,194 (9.6%)	323,516,147 (9.8%)	317914569 (11.5%)
DNA Transposons		61,865,922 (1.9%)	61,564,772 (1.9%)	61,401,094 (1.9%)	59,996,704 (1.8%)	61,763,643 (1.9%)	61284458 (2.2%)
Rolling Circles		290,862 (0.0%)	288,143 (0.0%)	292,224 (0.0%)	282,605 (0.0%)	289,875 (0.0%)	290924 (0.0%)
Unclassified		525,581 (0.0%)	479,986 (0.0%)	530,409 (0.0%)	468,391 (0.0%)	544,117 (0.0%)	560331 (0.0%)
Total Interspersed Repeats		1,349,379,752 (40.6%)	1,322,681,771 (40.8%)	1,328,314,056 (40.3%)	1,295,571,821 (39.7%)	1,337,618,166 (40.6%)	1279949819 (46.2%)
Satellites		475,498,670 (14.3%)	451,417,569 (13.9%)	491,232,699 (14.9%)	528,409,710 (16.2%)	467,096,074 (14.2%)	48578709 (1.8%)
Simple Repeats		27,545,432 (0.8%)	25,013,594 (0.8%)	26,856,901 (0.8%)	24,583,321 (0.8%)	27,251,561 (0.8%)	24028086 (0.9%)
Low Complexity		4,497,462 (0.1%)	4,273,074 (0.1%)	4,333,715 (0.1%)	4,256,759 (0.1%)	4,358,856 (0.1%)	4122310 (0.1%)
Small RNA		45,297,364 (1.4%)	44,731,650 (1.4%)	44,678,161 (1.4%)	43,653,726 (1.3%)	45,209,784 (1.4%)	43921056 (1.6%)
Total Repeats		1,858,696,960 (55.9%)	1,804,973,058 (55.7%)	1,852,269,687 (56.2%)	1,854,826,538 (56.9%)	1,838,123,418 (55.8%)	1357518521 (49.0%)

Percentages are calculated based on the total genome length of each assembly, and small summation discrepancies may occur due to overlapping annotations, or repeats classified in multiple categories by Repeatmasker.

**LINEs:** Long Interspersed Nuclear Elements; **SINEs**: Short Interspersed Nuclear Elements; **LTR:** Long Terminal Repeats, **PLEs:** Penelope-like Elements.

Among retroelements, LINEs (Long Interspersed Nuclear Elements) were the most abundant subtype, accounting for about 23–23.5%, followed by SINEs (Short Interspersed Nuclear Elements) at 9.6–9.9%, and LTR elements (Long Terminal Repeats) at 5.4–5.5%. Satellite DNA, a key component of centromeric and pericentromeric regions, displayed greater variability, ranging from 13.9% in Gudali to 16.2% in Singida White (50% of satellites are from unplaced contigs). Overall, the Mpwapwa breed had the highest repeat content in absolute terms, while Gudali had the lowest. Despite these differences, the general repeat composition and distribution patterns were broadly similar across all five cattle genomes, reflecting conserved repeat architecture among these breeds.

## Supplementary information

Shell scripts used to generate the genome assemblies

## Data Availability

The raw sequencing data generated in this study are available in the NCBI Sequence Read Archive (SRA) under BioProject accession PRJNA1197437 (SRA project SRP586484). Individual SRA accessions include SRX28862361, SRX28862362, SRX28862363, SRX28862364 and SRX28862365 corresponding to the Lagune, Gudali, Mpwapwa, Iringa Red and Singida White breeds, respectively. The scaffolded genome assemblies are also deposited under BioProject PRJNA1197437 and assigned the accession number JBNBOL000000000 with BioSample numbers SAMN45804670, SAMN45804671, SAMN45804672, SAMN45804673 and SAMN45804674^21^. Genome assemblies and associated files are additionally available via Figshare with https://doi.org/10.6084/m9.figshare.28840946.

## Code availability

Parameters for all the commands used to assemble and analyse the genomes were the default parameters of the corresponding software, unless mentioned and where necessary. The shell scripts detailing the exact sequence of commands used to generate the assemblies are provided in Supplementary 1.

## References

[1] di Lernia, S. *et al*. Inside the “African cattle complex”: Animal burials in the Holocene central Sahara. *PLoS One* **8**, e56879 (2013). 23437260 10.1371/journal.pone.0056879 PMC3577651

[2] Schneider, H. K. The subsistence role of cattle among the Pakot and in East Africa. *American Anthropologist* **59**, 278–300 (1957).

[3] Ajmone-Marsan, P., Lenstra, J. A. & Fernando Garcia, J. On the origin of cattle: how aurochs became domestic and colonized the world. *Evolutionary Anthropologist* **19**, 148–157 (2010).

[4] Mwai, O., Hanotte, O., Kwon, Y.-J. & Cho, S. African indigenous cattle: unique genetic resources in a rapidly changing world. *Asian-Australasian Journal of Animal Science* **28**, 911–921 (2015). 10.5713/ajas.15.0002R PMC4478499 26104394

[5] De Boer, H. Cattle genetic resources. *Livestock Production Science* **29**, 256–258 (1991).

[6] Talenti, A. *et al*. A cattle graph genome incorporating global breed diversity. *Nature Communications* **13**, 910 (2022). 35177600 10.1038/s41467-022-28605-0 PMC8854726

[7] Kim, K. *et al*. The mosaic genome of indigenous African cattle as a unique genetic resource for African pastoralism. *Nature Genetics* **52**, 1099–1110 (2020). 32989325 10.1038/s41588-020-0694-2

[8] Koren, S. *et al*. De novo assembly of haplotype-resolved genomes with trio binning. *Nature Biotechnology* **36**, 1174–1182 (2018). 10.1038/nbt.4277 PMC6476705 30346939

[9] Rosen, B. D. *et al*. De novo assembly of the cattle reference genome with single-molecule sequencing. *GigaScience* **9**, 1–9 (2020). 10.1093/gigascience/giaa021 PMC7081964 32191811

[10] Lloret-Villas, A. *et al*. Investigating the impact of reference assembly choice on genomic analyses in a cattle breed. *BMC Genomics* **22**, 363 (2021). 34011274 10.1186/s12864-021-07554-w PMC8132449

[11] Ahozonlin, M. C., Gbangboche, A. B., & Dossa, L. H. Current Knowledge on the Lagune Cattle Breed in Benin: A State of the Art Review. *Ruminants* **2**, 271-281.

[12] Zorobouragui, L. *et al*. Breeding practices and selection criteria in Gudali cattle breed from Benin: implications for the design of a community-based breeding program. *Frontiers in Animal Science* **5**, 1454071 (2025).

[13] Wilson, R. T. When is a “breed” not a breed: the myth of the Mpwapwa cattle of Tanzania. *Tropical Animal Health and Production* **53**, 233 (2021). 33783655 10.1007/s11250-021-02669-4

[14] Salum, K. A., Laswai, G. H. & Mushi, D. E. Performance of Boran and Two Strains of Tanzania Short Horn Zebu Cattle Fed on Three Different Diets. *International Journal of Animal Science and Technology* **8**, 21–29 (2024).

[15] Cheng, H., Concepcion, G. T., Feng, X., Zhang, H. & Li, H. Haplotype-resolved de novo assembly using phased assembly graphs with hifiasm. *Nature Methods* **18**, 170–175 (2021). 33526886 10.1038/s41592-020-01056-5 PMC7961889

[16] Alonge, M. *et al*. Automated assembly scaffolding using RagTag elevates a new tomato system for high-throughput genome editing. *Genome Biology* **23**, 258 (2022). 36522651 10.1186/s13059-022-02823-7 PMC9753292

[17] Chen, N. Using Repeat Masker to identify repetitive elements in genomic sequences. *Current protocols in bioinformatics* **5**, 4.10. 11–14.10. 14 (2004).

[18] Wheeler, T. J. *et al*. Dfam: a database of repetitive DNA based on profile hidden Markov models. *Nucleic Acids Research* **41**, D70–D82 (2013). 23203985 10.1093/nar/gks1265 PMC3531169

[19] Flynn, J. M. *et al*. RepeatModeler2 for automated genomic discovery of transposable element families. *Proceedings of the National Academy of Sciences* **117**, 9451–9457. 10.1073/pnas.1921046117 PMC7196820 32300014

[20] *NCBI Sequence Read Archive* https://identifiers.org/ncbi/insdc.sra:SRP586484 (2025).

[21] Houaga, I. *et al*. Genome assemblies of African cattle breeds using PacBio HiFi sequencing. *GenBank* https://identifiers.org/ncbi/insdc:JBNBOL000000000 (2026). 10.1038/s41597-026-07589-2 PMC13562511 42251053

[22] Ewels, P., Magnusson, M., Lundin, S. & Käller, M. MultiQC: Summarize analysis results for multiple tools and samples in a single report. *Bioinformatics* **32**, 3047–3048 (2016). 27312411 10.1093/bioinformatics/btw354 PMC5039924

[23] Ranallo-Benavidez, T. R., Jaron, K. S. & Schatz, M. C. GenomeScope 2.0 and Smudgeplot for reference-free profiling of polyploid genomes. *Nature Communications* **11**, 1432 (2020). 32188846 10.1038/s41467-020-14998-3 PMC7080791

[24] Rhie, A., Walenz, B. P., Koren, S. & Phillippy, A. M. Merqury: reference-free quality, completeness, and phasing assessment for genome assemblies. *Genome Biology* **21**, 245 (2020). 32928274 10.1186/s13059-020-02134-9 PMC7488777

[25] Huang, N. & Li, H. compleasm: a faster and more accurate reimplementation of BUSCO. *Bioinformatics* **39**, btad595 (2023). 37758247 10.1093/bioinformatics/btad595 PMC10558035

[26] Mikheenko, A., Prjibelski, A., Saveliev, V., Antipov, D. & Gurevich, A. Versatile genome assembly evaluation with QUAST-LG. *Bioinformatics* **34**, i142–i150 (2018). 29949969 10.1093/bioinformatics/bty266 PMC6022658

[27] Katz, L. S. *et al*. Mashtree: a rapid comparison of whole genome sequence files. *Journal of Open-Source Software* **4**, 1762 (2019). 10.21105/joss.01762 PMC9380445 35978566

