# Supplementary material for: High quality genome assemblies of African cattle breeds using PacBio HiFi sequencing
Source: Sci Data. 2026 Jun 6;13:1300. doi: 10.1038/s41597-026-07589-2 (PMC13562511; doi:10.1038/s41597-026-07589-2)
Supplement: Supplementary file 1 [file 41597_2026_7589_MOESM1_ESM.docx]

#!/bin/sh

\# SGE options (lines prefixed with #$)

#$ -N HIFIASM

#$ -cwd

#$ -pe sharedmem 12

#$ -l h_rt=96:00:00

#$ -l h_vmem=20G

#$ -P roslin_HighlanderLab

```
## 1. Create a text file with fast files and id name
## echo "`ls /exports/cmvm/eddie/eb/groups/Highlander_African5/Lagune_Benin/fastq_lagune/* | tr "\n" " " `LAG" >> fastq_path_ids.txt
## 3. and submit them via following command
## qsub -t 1:5 hifiasm_new.sh fastq_path_ids.txt
```

\# Initialise the environment modules

. /etc/profile.d/modules.sh

\# Read the specified line and split into variables

awk -v line="$SGE_TASK_ID" 'NR == line {print $1, $2, $3}' $1 | while read -r pathid; do

fastq_files=$(echo "$pathid" | awk '{print $1" "$2}')

id=$(echo "$pathid" | awk '{print $3}')

echo "fastq: $fastq_files"

echo "ID: $id"

/exports/cmvm/eddie/eb/groups/Highlander_African5/Meenu/Tools/hifiasm/hifiasm -z20 -o ${id}_trim.asm -t 12 $fastq_files

done
